# Reply to Yu et al.: Meteorological covariations do not reproduce diurnal cloud fraction response to aerosol

**DOI:** 10.1073/pnas.2616376123

**Published:** 2026-07-02

**Authors:** Geoffrey Pugsley, Edward Gryspeerdt, Vishnu Nair

**Affiliations:** ^a^ Department of Physics, Imperial College London, London SW7 2BW, United Kingdom

We thank Yu et al. (1) for their interest in our work and for raising an important point about the possible impacts of meteorological confounders when assessing aerosol–cloud relationships. Pugsley et al. (2) focused on the cloud fraction (CF) evolution, adjusted (∆CF*_adj_*) to account for known covariations between aerosol and CF (3), together with a corresponding analysis for LWP. While Yu et al. (1) show that it is statistically possible for additional confounders to exist (as in any observed relationship), the lack of a clear physical mechanism for any of the proposed confounders makes it unlikely they are responsible for the results observed.

**Fig. 1.**
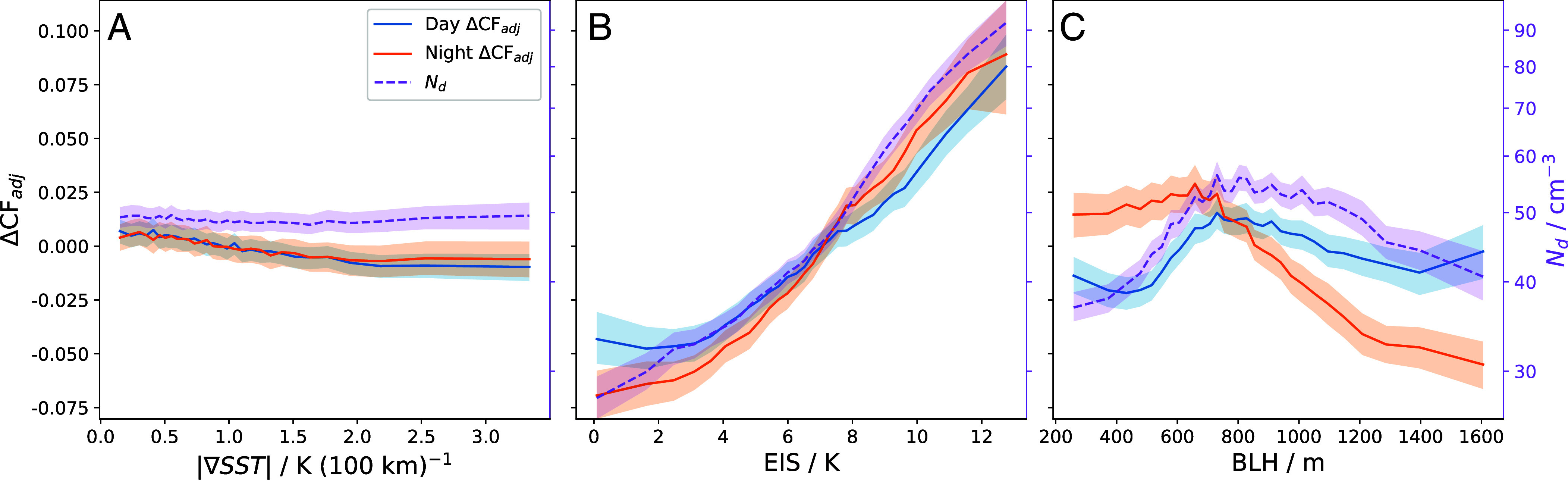
Mean *N_d_* at the first Terra overpass, and ∆CF*_adj_* along the trajectories as a function of *∇*SST (sea-surface temperature gradient; (*A*) EIS (estimated inversion strength; (*B*) and BLH (boundary layer height; (*C*) evaluated at trajectory initialization (06:00 LST), illustrating the degree of meteorological covariability with both quantities. Shading denotes the 95% CI estimated using bootstrap resampling with 1,000 samples.

**Fig. 2.**
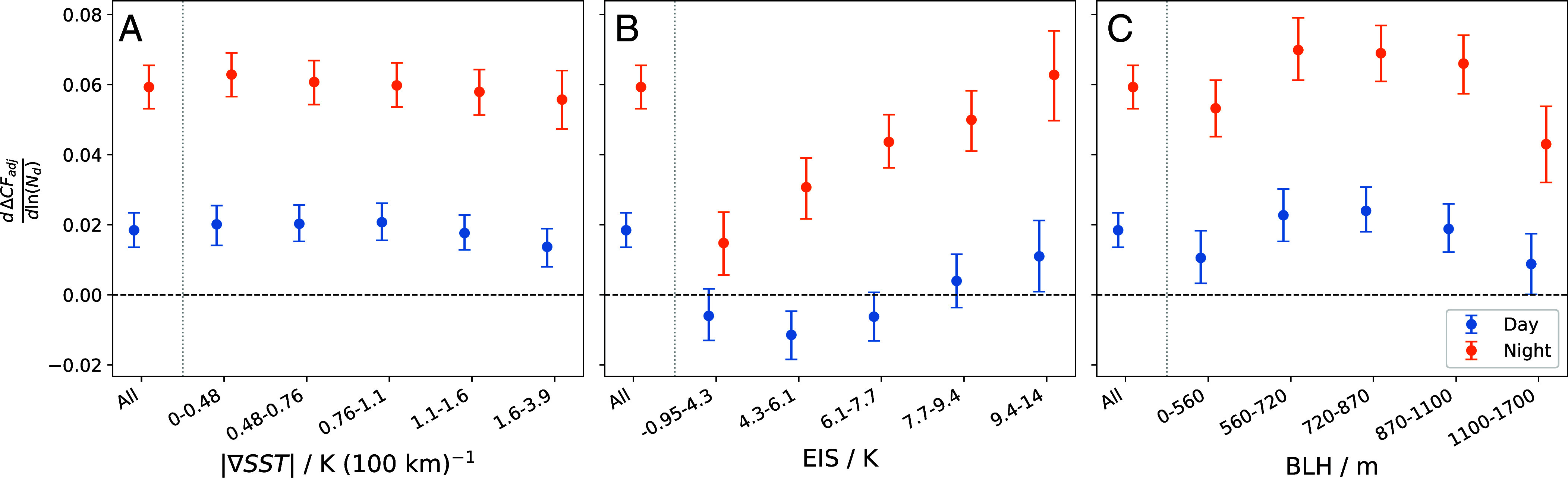
Linear regression slope of ∆CF*_adj_* against ln(*N_d_*), stratified into meteorological quintiles of *∇*SST (*A*), EIS (*B*), and BLH (*C*) at the time of trajectory initialization, with each regime containing an equal number of trajectories. The qualitative trends remain robust to the choice of meteorological binning; in particular, stratifying by *∇*SST and BLH has no significant impact on the slope. For EIS (*B*), the strongest gradients occur in the most stable regimes, consistent with enhanced aerosol susceptibility in stable stratocumulus environments (7). The regression slope over all the trajectories is included on the left-hand side of each panel for reference. Error bars denote 95% CI estimated as in Fig. 1.

A potential confounder must satisfy the following conditions (at a minimum):

1. There must be a physical pathway for the confounder to control both *N_d_* and ∆CF*_adj_*. While SST gradients have been shown to be related to stratocumulus cloud breakup (4), the mechanism for an impact on *N_d_* is unclear. We find no relationship between SST gradient and *N_d_* (Fig. 1*A*), and it is hence unable to produce the observed *N_d_* –∆CF*_adj_* relationships.
2. The meteorological relationship with ∆CF*_adj_* must itself vary systematically between day and night to explain the weaker daytime than nighttime *N_d_* –∆CF*_adj_* relationship demonstrated in ref. 2. Although EIS has a known strong relationship with CF (5) and clear relationships with *N_d_* and ∆CF*_adj_*, its relationship with ∆CF*_adj_* shows very weak diurnal variation (Fig. 1*B*). EIS therefore fails this test.
3. The proposed confounder must explain the temporal aspect of the *N_d_* –∆CF*_adj_* relationship. The *N_d_* data in our work come from the start of the three-day trajectories, such that it is not temporally coincident with the ∆CF*_adj_* observations. While the instantaneous *N_d_* -BLH relationship is well supported by theoretical considerations (6), for BLH to act as a confounder in this work, it would need to impact both *N_d_* and subsequent ∆CF*_adj_* over multiday timescales, independently of the initial CF (as it is removed by the definition of CF*_adj_*), making such a mechanism unlikely.
4. Conditioning on a confounding meteorological variable must substantially weaken or remove the *N_d_* – ∆CF*_adj_* relationship. The enhanced nighttime *N_d_*–∆CF*_adj_* relationship persists within all meteorological regimes (Fig. 2 *A*–*C*), demonstrating that the observed relationship is not explained by the meteorological covariations suggested by Yu et al. (1).

Understanding meteorological confounding remains an important challenge in observational studies of aerosol–cloud interactions. By removing the impact of the initial CF, ∆CF*_adj_* reduces the role of meteorological covariations. The analyses presented here demonstrate that while the proposed meteorological confounders modulate the relationship observed in Pugsley et al. (2), particularly through EIS (Fig. 2*B*), they cannot reproduce the observed day–night contrast.

In addition, the diurnal variation observed in Pugsley et al. (2) is consistent with model (8) and observational studies (9) finding a diurnal variation in aerosol–cloud interactions. This would be physically consistent with precipitation suppression mechanisms (10) acting most effectively during nighttime when stratocumuli are most likely to be precipitating (11).
